# Long-range dependence in earthquake-moment release and implications for earthquake occurrence probability

**DOI:** 10.1038/s41598-018-23709-4

**Published:** 2018-03-28

**Authors:** Simone Barani, Claudia Mascandola, Eva Riccomagno, Daniele Spallarossa, Dario Albarello, Gabriele Ferretti, Davide Scafidi, Paolo Augliera, Marco Massa

**Affiliations:** 1 Dipartimento di Scienze della Terra dell’Ambiente e della Vita, Università degli Studi di Genova, Genova, Italy; 2 Istituto Nazionale di Geofisica e Vulcanologia, Sezione di Milano, Milano, Italy; 3 Dipartimento di Matematica, Università degli Studi di Genova, Genova, Italy; 4 Dipartimento di Scienze Fisiche della Terra e dell’Ambiente, Università degli Studi di Siena, Siena, Italy

## Abstract

Since the beginning of the 1980s, when Mandelbrot observed that earthquakes occur on ‘fractal’ self-similar sets, many studies have investigated the dynamical mechanisms that lead to self-similarities in the earthquake process. Interpreting seismicity as a self-similar process is undoubtedly convenient to bypass the physical complexities related to the actual process. Self-similar processes are indeed invariant under suitable scaling of space and time. In this study, we show that long-range dependence is an inherent feature of the seismic process, and is universal. Examination of series of cumulative seismic moment both in Italy and worldwide through Hurst’s rescaled range analysis shows that seismicity is a memory process with a Hurst exponent *H* ≈ 0.87. We observe that *H* is substantially space- and time-invariant, except in cases of catalog incompleteness. This has implications for earthquake forecasting. Hence, we have developed a probability model for earthquake occurrence that allows for long-range dependence in the seismic process. Unlike the Poisson model, dependent events are allowed. This model can be easily transferred to other disciplines that deal with self-similar processes.

## Introduction

Spatial and temporal correlations, self-similarities, and event interactions are common features of natural phenomena that exhibit self-organized criticality. Since the seminal work of Bak *et al*.^1^, many studies have interpreted seismicity as the result of a dynamical process exhibiting a self-organized critical behavior^2–6^. The Earth crust, subjected to the driving force from tectonic plate motion, is assumed to be a dissipative dynamic system that naturally self-organizes into a stationary state, which is critical (i.e., metastable). A stationary state can be viewed as a critical chain reaction. When an earthquake occurs, stress perturbations propagate through the crust and, similarly to the ‘domino’ effect, upset neighboring and distant zones. These, in turn, release earthquakes when the total accumulated stress exceeds the friction force. This chain process is self-organized and can continue indefinitely.

As with many stochastic processes that exhibit self-organized criticality (e.g., avalanches, rainstorms, landscape evolution, drainage network dynamics, stock prices, Ethernet traffic, and gene expression dynamics), the spatial and temporal scaling of earthquakes obeys power law distributions. One of the fundamental scaling laws in seismology is the magnitude-frequency distribution of earthquakes^7^. This law arises from the self-similarity in the fracturing process^8–11^. Along with the space-time clustering of seismicity^12,13^ and the mechanics of earthquake interaction^11,14^, this property implies that the process of accumulation and release of seismic strain has memory effects^15^. Periods of high release of seismic deformation will most likely be followed by years of higher-than-average seismic strain release. Conversely, seismically quiet periods will tend to be followed by quiet years. This behavior mimics that of fluctuating long-term water storage in reservoirs. Tectonic loading and seismic strain release have the roles of the inflow and outflow, respectively. Hence, we expect a certain degree of correlation in time series of seismic strain release.

To study fluctuations in stochastic processes, Hurst^16^ introduced rescaled range (*R*/*S*) analysis^17,18^. *R*/*S* analysis splits a time series into *N* adjacent (generally non-overlapping) windows of size *n*, and inspects the range *R* of the fluctuations (with respect to an average trend), rescaled by the sample standard deviation *S*, for different values of *n* (here taken as positive integer). Precisely, for all subseries of length *n*, the rescaled range analysis calculates the mean value of the rescaled range (*R*/*S*)_*n*_ as:

1 $${(R/S)}_{n}=\frac{1}{N}\sum _{k=1}^{N}\frac{R{(n)}_{k}}{S{(n)}_{k}}$$

where *R*(*n*)_*k*_ is the range from maximum to minimum of the cumulative deviations calculated in the *k*-th sub-interval of length *n*, *S*(*n*)_*k*_ is the sample standard deviation, and *R*(*n*)_*k*_/*S*(*n*)_*k*_ is the *k*-th rescaled range. A specific mathematical definition of *R*(*n*)_*k*_ and *S*(*n*)_*k*_ is provided in the Method section, while it is important to emphasize here the physical meaning of *R*. Hurst^16,19^ defined *R* as the storage capacity that allows the mean discharge of a stream to be maintained over a number of years. Hence, *R* is representative of the maximum (or critical) capacity of a reservoir for a period of length *n* (e.g., *n* years). With reference to the process of seismic strain accumulation and release, *R* can therefore be interpreted as the maximum seismic capacity, which is defined as the greatest amount of elastic energy that can be stored (or released) by a seismogenic source over a certain time period. Hence, this can provide a conservative estimate for the maximum earthquake magnitude for the period^15^. Alternatively, it can be viewed as the critical capacity associated with a time interval of size *n*, above which a maximum release of deformation might occur.

Hurst found that the rescaled range (*R*/*S*)_*n*_ is empirically related to *n* by a power law with exponent *H*, known as the Hurst exponent:

2 $${(R/S)}_{n}\, \sim c{n}^{H}$$

The Hurst exponent measures the level of correlation (memory) in time series. It takes values between 0 and 1 and indicates a persistent behavior (or super-diffusive process) if *H* > 0.5, and anti-persistent behavior if *H* < 0.5 (or sub-diffusive process). A value close to 0.5 indicates a random process with no correlation and no dependence within the series. Many natural phenomena show persistent behaviors, with Hurst exponent generally fluctuating around 0.73^20^. This holds true also for earthquakes^21–28^, even though some variability in the *H* value has been documented^6,29–31^. However, most of these studies focused on specific seismic episodes (e.g., seismic sequences) or examined the process taking into account short observation periods. Moreover, some of them analyzed series of magnitude, inter-event times, or earthquake frequency rather than of seismic moment or energy. Hence, the maximum deviation *R* loses the physical interpretation originally given by Hurst^15^.

In the present study, we examine series of seismic moment release through Hurst’s *R*/*S* analysis to determine whether and how the degree of correlation among earthquakes in the series (expressed by *H*) varies both spatially and temporally (i.e., as a function of the length of the seismic record). We analyze time series of cumulative seismic moment for single sites and extended areas in the Italian Central Apennines, accounting for the entire historic record and focusing on specific seismic episodes. The Italian Central Apennines are among the regions with the highest seismic hazard in the Mediterranean and central-western Europe^32^. Strong earthquakes have occurred both in the past and in recent years, when the three major seismic crises that are examined in the following shocked the population, between 1997 and 2017. The values of the Hurst exponent in this region are then compared with those obtained for all of Italy and worldwide, to reveal the universal value of *H*. We will show that variations in the *H* value are actually fictitious, as they are caused by the limited extent of earthquake time series or by catalog incompleteness, particularly at larger magnitudes.

Following the results of the *R*/*S* analysis, which confirm that seismicity is a memory process that is characterized by long-range dependence, we present a new forecasting model that allows for persistency in the release of seismic deformation. The scientific literature includes many different kinds of earthquake forecasting models that are based on different assumptions and have different scopes^33–41^. While some models are developed for short-term forecasting (i.e., days to weeks), others are more suitable for medium-term forecast (i.e., months to a few years), and some others aim at forecasting earthquakes in the long term (i.e., to tens of years). This last group includes models that are at the core of probabilistic seismic hazard analysis (PSHA), the primary tool for anti-seismic design and emergency-response planning^42^. Nowadays, time-independent models based on the assumption of seismicity as a Poisson process are still widely used worldwide in PSHA. The key to the success of the Poisson model for earthquake occurrence in both hazard and risk analyses mostly relies on its simplicity. Although various models have been proposed as an alternative to the Poisson model, most of them have limited applicability due their extensive parameterization, which is often accompanied by lack of the experimental data (e.g., observations of repeated events on individual faults^43,44^) required to estimate the model parameters.

In the present study, we focus primarily on long-term forecasting. However, this does not imply that the model proposed is limited to applications in the long term only. In more detail, the scope is to develop a model that consistent with the results of the *R*/*S* analysis: (1) incorporates long-range dependence in the seismic process; (2) is reliable (i.e., forecasts are compatible with observations); and (3) does not require extensive parameterization, thus allowing the widest possible applicability. To accomplish this third item, our model does not assume any a-priori statistical distribution of earthquake inter-event times, nor any specific hypothesis on the process of accumulation and release of stress on faults. In other words, we leave the catalog data ‘speak’ for themselves.

## Results

### Universality of the Hurst exponent

We present the results both in terms of maps and diagrams that show the temporal variation of *H* for single sites. The diagrams are obtained by keeping the starting time fixed while moving the ending time forward 1 year at a time, and plotting the data points at the end of each time interval. The procedure adopted to determine the time series analyzed through the *R*/*S* analysis is described in the Method section.

**Figure 1:**
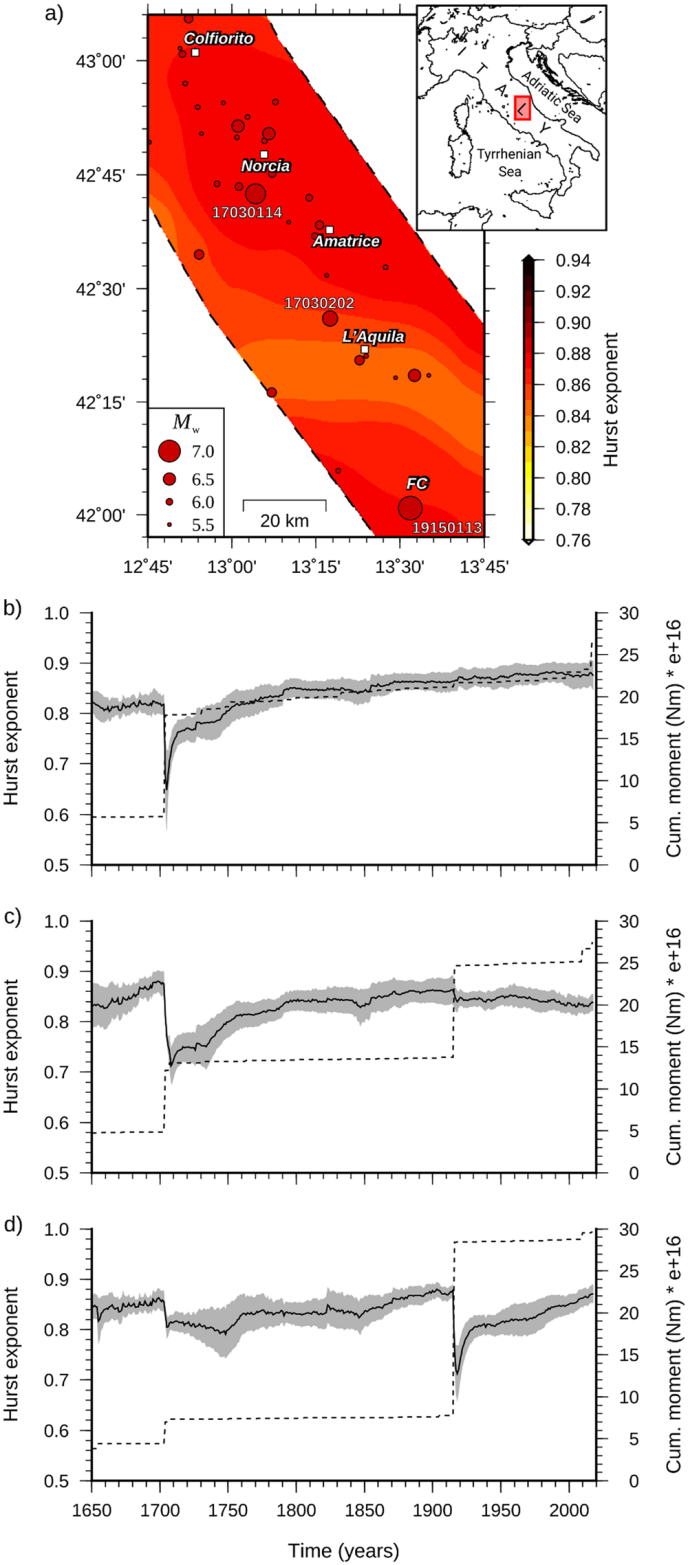
Up-to-date geographic distribution of the Hurst index in central Italy (**a**) and temporal variations for the sites of Norcia (**b**), L’Aquila (**c**), and in the Fucino (FC) basin (**d**). The date of the largest shocks that occurred in the area are displayed in Fig. 1a. The shaded areas in Fig. 1b–d indicate the 68% confidence interval. The time series of cumulative seismic moment are superimposed in Fig. 1b–d (dashed lines).

**Figure 2:**
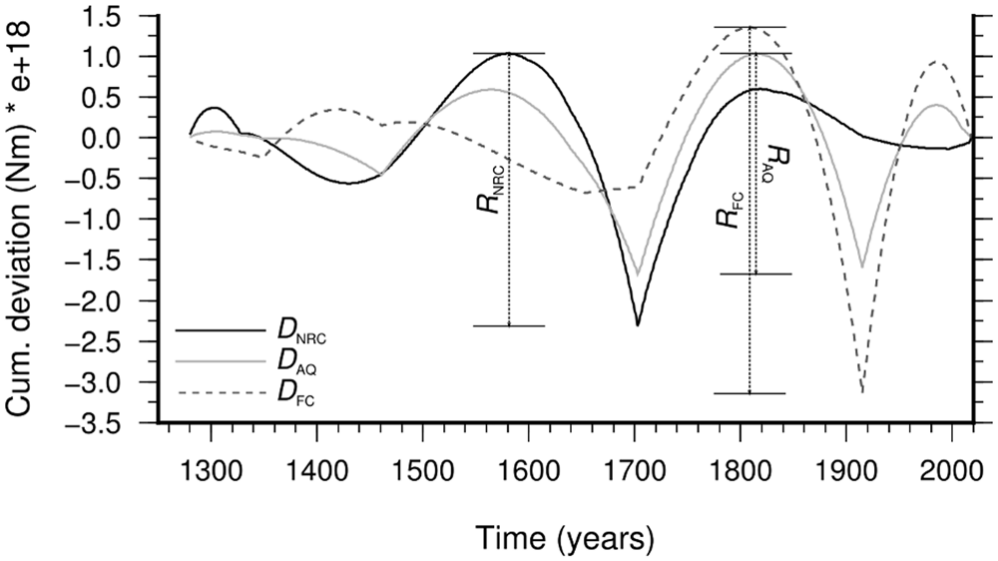
Comparison of the cumulative deviation functions for *N* = 1 (see equation (9) in the Method section) for Norcia (NRC), L’Aquila (AQ), and a site in the Fucino (FC) basin. The figure shows that *R*_AQ_ < *R*_NRC_ < *R*_FC_, where *R* indicates the range from maximum to minimum of the cumulative deviations.

Figure 1a illustrates the geographic distribution of the Hurst index in central Italy. Input time series were determined from the Parametric Catalogue of Italian Earthquakes 2015 – CPTI15^45^ updated to February 2017 with the data provided by the INGV Centro Nazionale Terremoti (http://cnt.rm.ingv.it/). The map clearly reveals little spatial variability in the *H* value. Indeed, *H* ranges between 0.86 and 0.88, which indicates a marked persistence in the process of seismic moment release (i.e., *H* ≫ 0.5). Slightly lower values (around 0.84) that, however, fall within the uncertainty bounds of *H* (Fig. 1b–d, shaded areas), can be observed all over a corridor that cuts across the Central Apennines through L’Aquila, where *R* assumes a lower value (Fig. 2). This might be indicative of a lower seismic capacity, although it might be affected by the detrending polynomial used in the computation of *R* (see Method section) and by the completeness of the catalog in this area, where the largest events might occur with very long recurrence times^46^. Despite its extent (1000 – February 2017), indeed, the catalog might be too short to include some of the largest events that this sector of the Central Apennines can produce. This might affect the time series trend and, albeit to a lesser extent, the value of the Hurst exponent.

**Figure 3:**
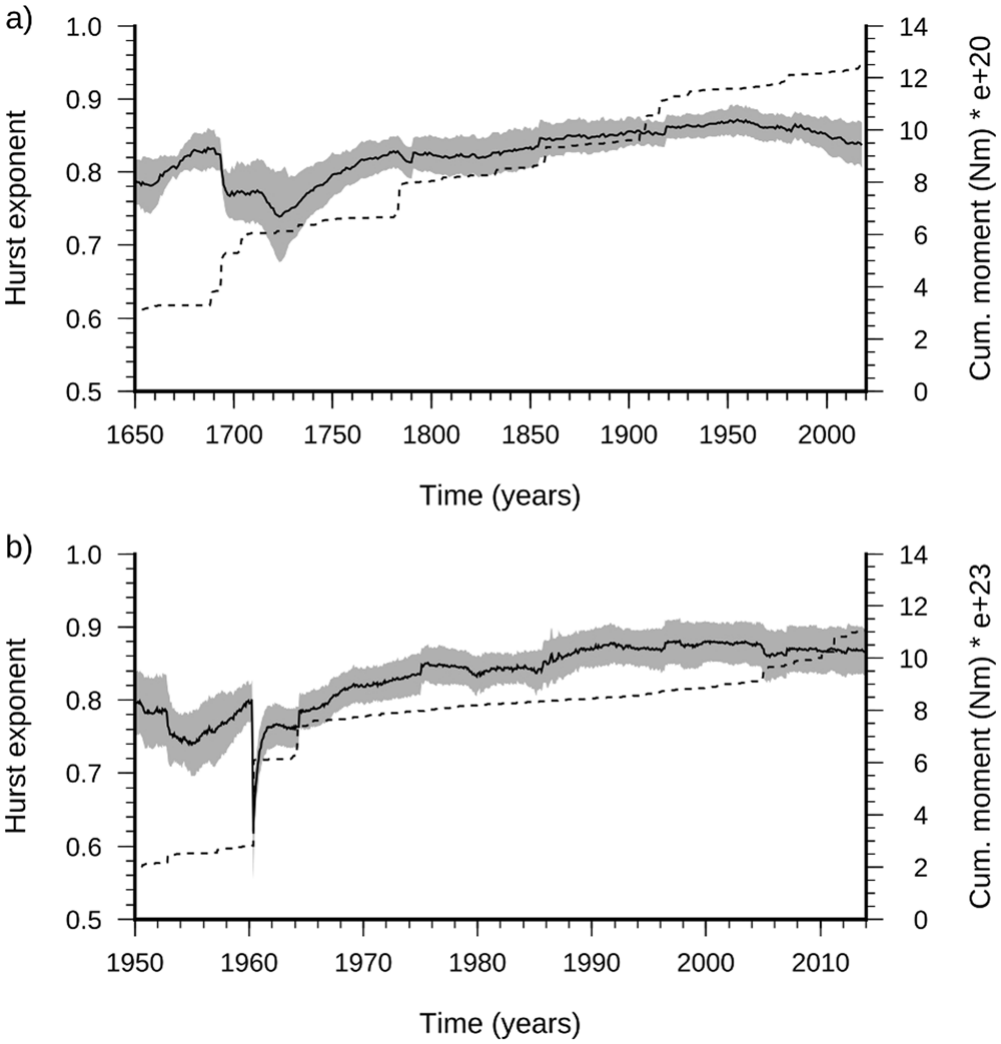
Temporal variation of the Hurst exponent for the Italian (**a**) and worldwide (**b**) seismicity. The time series of cumulative seismic moment are superimposed (dashed lines). The Italian seismicity is from the CPTI15 catalog^45^ updated to 2017. Worldwide seismicity is from the ISC-GEM catalog^47^.

For the temporal variability of the Hurst exponent (Fig. 1b–d), our analysis reveals that *H* is substantially time invariant, with sharp fluctuations associated only with the largest earthquakes that occurred in the study area. These events correspond to the 1703 seismic sequence between Norcia and L’Aquila (with three consecutive events, two of which had *M*_w_ 6.7 and 6.9), and the 1915 Fucino (FC) earthquake of *M*_w_ = 7.1. These spurious fluctuations are attributable to catalog incompleteness. Following these major shocks, indeed, the Hurst coefficient regains the original value and remains insensitive to the subsequent strong events. Only an exceedance of the maximum observed value of *R* will produce a new drop in *H*. The time invariance of the Hurst exponent is confirmed by the analysis of the Italian and worldwide seismicity (Fig. 3), which leads to *H* values similar to those for the Central Apennines. Of particular note, there is the drop produced by the 1960 Valdivia earthquake, Chile, with *M*_w_ = 9.6 (Fig. 3b), which is again attributable to the limited extent (1900–2013) of the catalog^47^.

**Figure 4:**
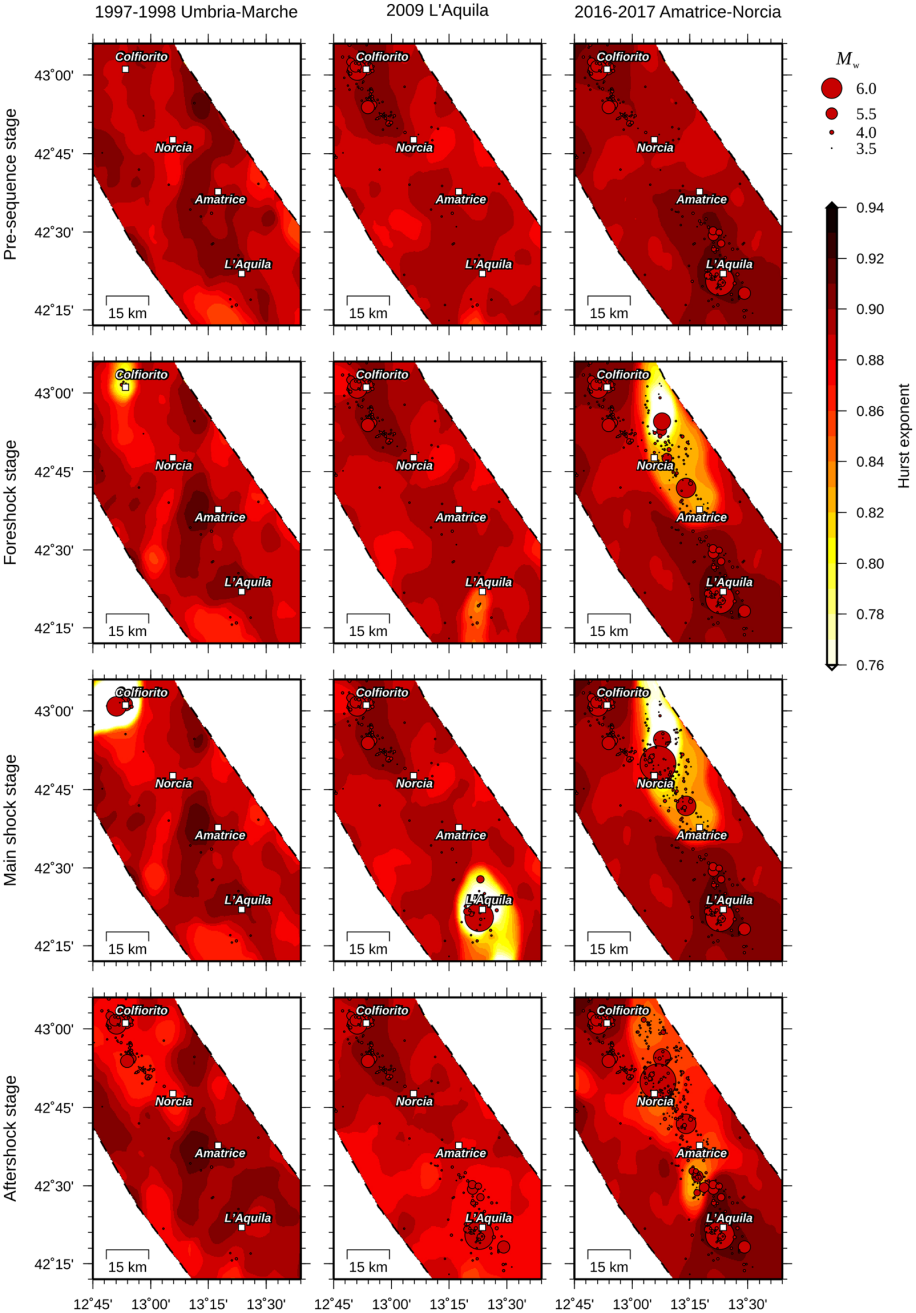
Geographic distribution of the Hurst index before (pre-sequence stage) and during (foreshock, main shock, and aftershock stages) the 1997–1998 Umbria-Marche, 2009 L’Aquila, and 2016–2017 Amatrice-Norcia seismic sequences.

The foregoing observations suggest the invariance of *H* both temporally and spatially. However, similarly to the Gutenberg and Richter *b*-value, fictitious changes (i.e., not related to the physical process of strain release) in the *H* value might be observed on short time scales. In this regard, three case studies that correspond to different seismic sequences in the Central Apennines are illustrated (1997–1998 Umbria-Marche; 2009 L’Aquila; 2016–2017 Amatrice-Norcia). In these examples, only the instrumental seismicity recorded since 1981 is taken into account (http://cnt.rm.ingv.it/). Figure 4 shows snapshot maps for each of these, which display the spatial distribution of *H* at different stages; namely, the pre-sequence, foreshock, main shock, and aftershock stages. Since the first stage, the sequences take place in areas generally characterized by lower values of *H*. With the foreshock occurrence, *H* begins to decrease and the nucleation zone – that is, the area where the main shock will occur and the sequence will start evolving – becomes distinguishable. The extent of the nucleation zone depends on both the foreshock distribution and energy. It is clearly wider in the case of the Amatrice-Norcia sequence, which was characterized by two strong foreshocks of *M*_w_ between 5.9 and 6. The *H* coefficient reaches the minimum value following the main shock occurrence and regains the base value during the subsequent months or years. According to previous findings, the variations of the *H* value discussed in these three case studies are undoubtedly due to the short time period covered by the earthquake catalog, which is evidently incomplete for larger magnitudes.

**Figure 5:**
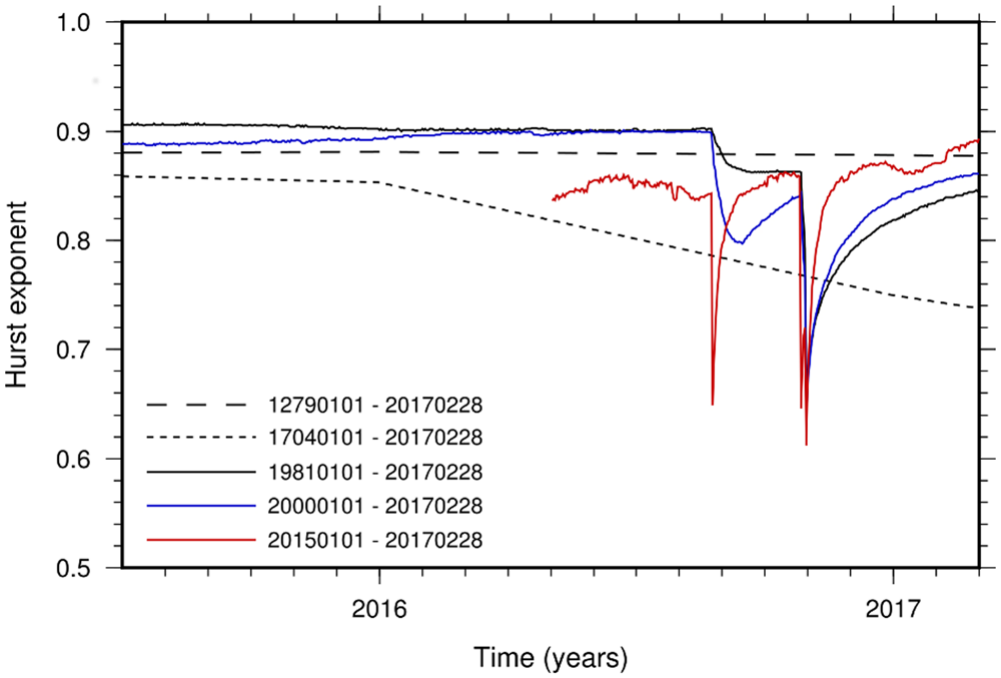
Temporal variation of the Hurst exponent before and during the 2016–2017 Amatrice-Norcia sequence for different earthquake catalogs with different extents and levels of completeness. Yearly variations are examined in the case of historical seismicity (dashed lines) while daily variations are considered in the analysis of the recent seismicity.

Figure 5 illustrates the sensitivity of *H* to moment release as a function of catalog completeness for the site of Norcia. Specifically, it shows the temporal variation of *H* before and during the 2016–2017 Amatrice-Norcia sequence for different earthquake catalogs with different temporal extents and minimum magnitude threshold, *m*_min_. Besides the catalogs adopted for the examples displayed in Figs 1 and 4 (with *m*_min_ = 3.5_,_ and *m*_min_ = 0, respectively), we analyzed an instrumental dataset that collects the earthquakes recorded in the area shocked by the sequence since January 2015 with magnitudes as low as *m*_min_ = −1.7. The number and amplitude of the drops marked on each line and the extent of the restoration stages measure the sensitivity of *H* to moment release. As expected, the sensitivity of *H* is inversely proportional to catalog completeness. The longer the seismic history (i.e., greater completeness for larger magnitudes), the higher the stability of *H*. Drops in the *H* value become sharper and the restoration stages shorter as the record length reduces. The two-year instrumental data set (Fig. 5, red line) allows clear identification of two fully restored drops, which correspond to the August 24 *M*_w_ = 6 foreshock and the October 30 *M*_w_ = 6.5 main shock. A small indentation is also discernible in the second drop, which corresponds to the October 26 *M*_w_ = 5.9 foreshock. The *H* variations become increasingly less evident as the catalog completeness increases, and they disappear when the entire historic record is considered. If this latter is constrained back to 1704, thus removing part of the seismic history and the contribution of the 1703 event, then the effect of the 2016 Amatrice-Norcia sequence appears, with a mild reduction of the Hurst exponent.

### Incorporating long-range dependence into forecasting models

Hurst index values such as those discussed above allow us to confirm that seismicity is a memory process with long-range dependence. This invalidates the assumption of seismicity as a Poisson process, which is widely used in conventional PSHA, and motivates the development of a probability model for earthquake occurrence that allows for persistency in the release of seismic energy.

**Figure 6:**
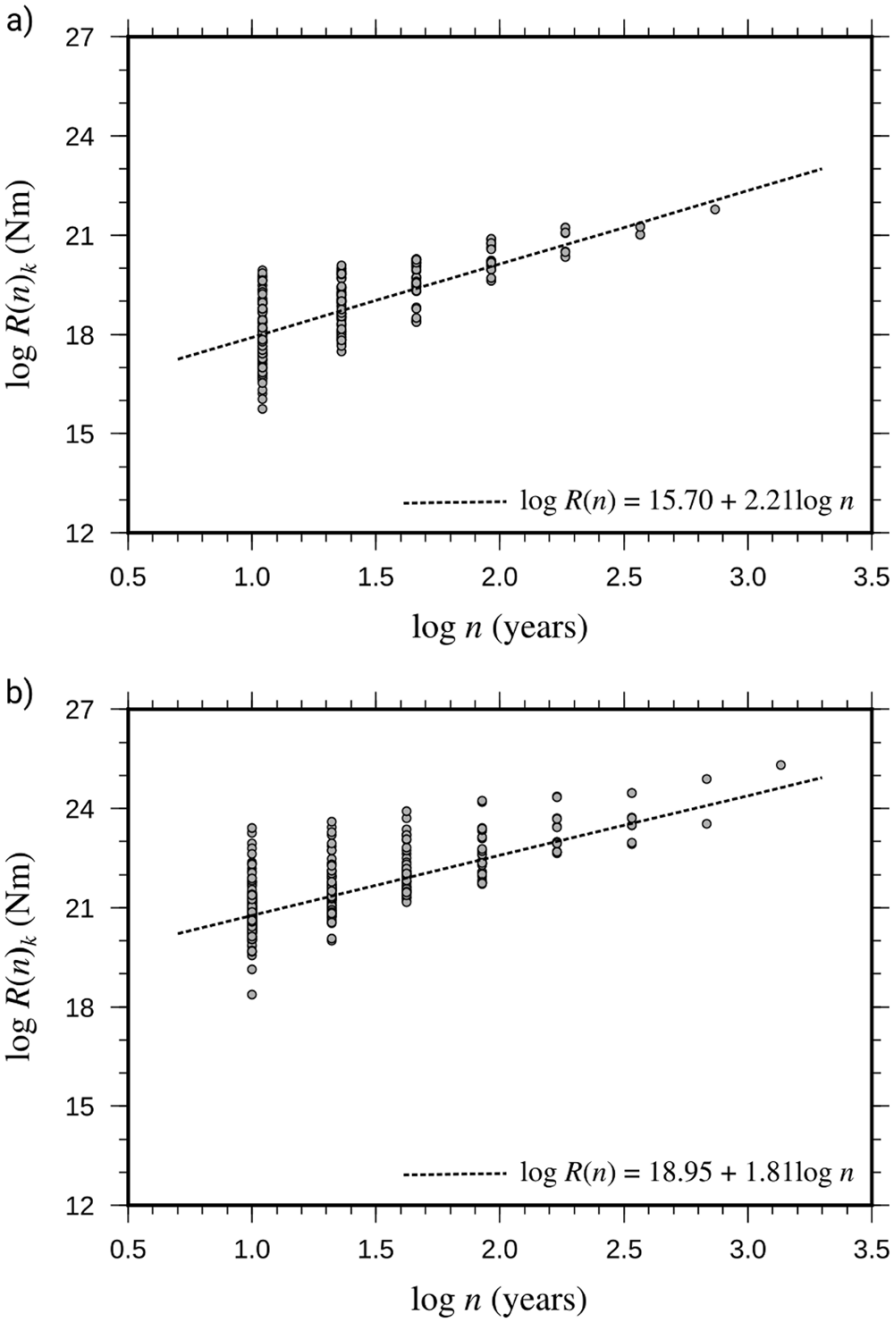
Scatter plot of log*R*(*n*)_*k*_ versus log*n* obtained from the Italian (**a**) and worldwide (**b**) time series shown in Fig. 3. For each value of the time length *n*, the logarithmic range is plotted for the available sub-intervals indexed by *k*. The slope of the regression lines is the estimated *K* exponent (equation (3)).

The simplest way to incorporate the previous findings into probability models for earthquake occurrence is to derive an empirical predictive relation for *R*, as is representative of the seismic source potential. We recall from the Introduction that *R* estimates the critical seismic moment, ${M}_{0}^{c}$ (where *M*_0_ indicates conventionally the scalar moment and the superscript *c* stands for critical), corresponding to a time period of length *n*. Fig. 6 shows the scatter plot of log*R*(*n*)_*k*_ versus log*n* for the Italian and worldwide data sets. The increasing trend in Fig. 6 can be estimated by least-squares regression, and yields the definition of *R*(*n*) as the predicted value of *R* at *n*; namely:

3 $$R(n) \sim d{n}^{K}$$

where *K* and *d* are region dependent (see Fig. 6 and Table S1 in the Supplementary Note).

Replacing *R*(*n*) with ${M}_{0}^{c}(n)$ and *R*(*n*)_*k*_ with ${M}_{0}^{c}{(n)}_{k}$, equation (3) can be written as:

4 $$\mathrm{log}\,{M}_{0}^{c}{(n)}_{k}=q+K\,\mathrm{log}\,n+{\xi }_{\mathrm{log}{M}_{0}^{c}{(n)}_{k}}$$

where $q=\,\mathrm{log}\,d$, and ${\xi }_{\mathrm{log}{M}_{0}^{c}{(n)}_{k}}$ is the Gaussian residual with zero mean and standard deviation ${\sigma }_{\mathrm{log}{M}_{0}^{c}(n)}$.

Equation 4 is a predictive model for $\mathrm{log}\,{M}_{0}^{c}{(n)}_{k}$, where $q+K\,\mathrm{log}\,n$ represents the theoretical mean of the logarithm of the random variable ${M}_{0}^{c}(n)$. It can be shown that the previous relation incorporates long-range dependence in the process of seismic strain release. Indeed, as shown in the Supplementary Note, the value of *K* can be recovered from *H* as:

5 $$K\approx H+(\frac{\mathrm{log}\,S(n)+p-q}{\mathrm{log}\,n})$$

where $p=\,\mathrm{log}\,c$ (see equation (2) for the meaning of *c*), and log*S*(*n*) can be determined via linear regression; namely, by assuming that *S*(*n*) also follows a power law.

We can compute the probability of exceedance of a specified moment value ${M}_{0}^{\ast }$ in a given time period Δ*t* = *n* (e.g., *n* years) as:

6 $$P({M}_{0}^{c}(n) > {M}_{0}^{\ast }|{\rm{\Delta }}t)=1-{F}_{{M}_{0}^{c}(n)}({M}_{0}^{\ast })$$

where ${F}_{{M}_{0}^{c}(n)}$ is the cumulative distribution function (CDF) of ${M}_{0}^{c}(n)$.

To compute the probability of exceeding a target ${M}_{0}^{\ast }$ value, the probability distribution of ${M}_{0}^{c}(n)$ has to be specified. To this end, we performed a Kolmogorov-Smirnov test on a number of empirical CDFs of ${M}_{0}^{c}(n)$ that were obtained from the Italian and worldwide cumulative moment series, as well as from some local series in the Italian Central Apennines. We assumed the null hypothesis that ${M}_{0}^{c}(n)$ is lognormally distributed, and a conventional 5% significance level. The results (not shown here for the sake of brevity) showed that the data are drawn from the expected lognormal distribution, which is therefore assumed in the probability computations presented in the example below. However, other heavy-tailed distributions can be adopted. Note, indeed, that heavy-tailed distributions are commonly used to model fractal phenomena^8^.

Given the Hanks and Kanomori^48^ relation between *M*_w_ and *M*_0_, the probability of exceeding a target ${M}_{0}^{\ast }$ value in Δ*t* years translates into the probability of exceeding the magnitude value corresponding to ${M}_{0}^{\ast }$. Alternatively, the probability of exceedance of a target magnitude value *m* can be obtained as the probability of exceeding the corresponding ${M}_{0}^{\ast }$ value; namely:

7 $$P({M}_{{\rm{w}}} > m)=P({M}_{0}^{c}(n) > {10}^{1.5m+9.1}|{\rm{\Delta }}t)=1-{F}_{{M}_{0}^{c}(n)}({10}^{1.5m+9.1})$$

Therefore, equation (4) and equation (7) can be conservatively used to determine the probability of at least one exceedance of a particular earthquake magnitude in a specified time interval. The word conservatively is used because *R* is representative of the critical (or maximum) seismic capacity. However, a single maximum earthquake that releases the total accumulated energy might never occur. On the other hand, the maximum stored energy might be released by a main shock with its foreshocks, and aftershocks.

**Figure 7:**
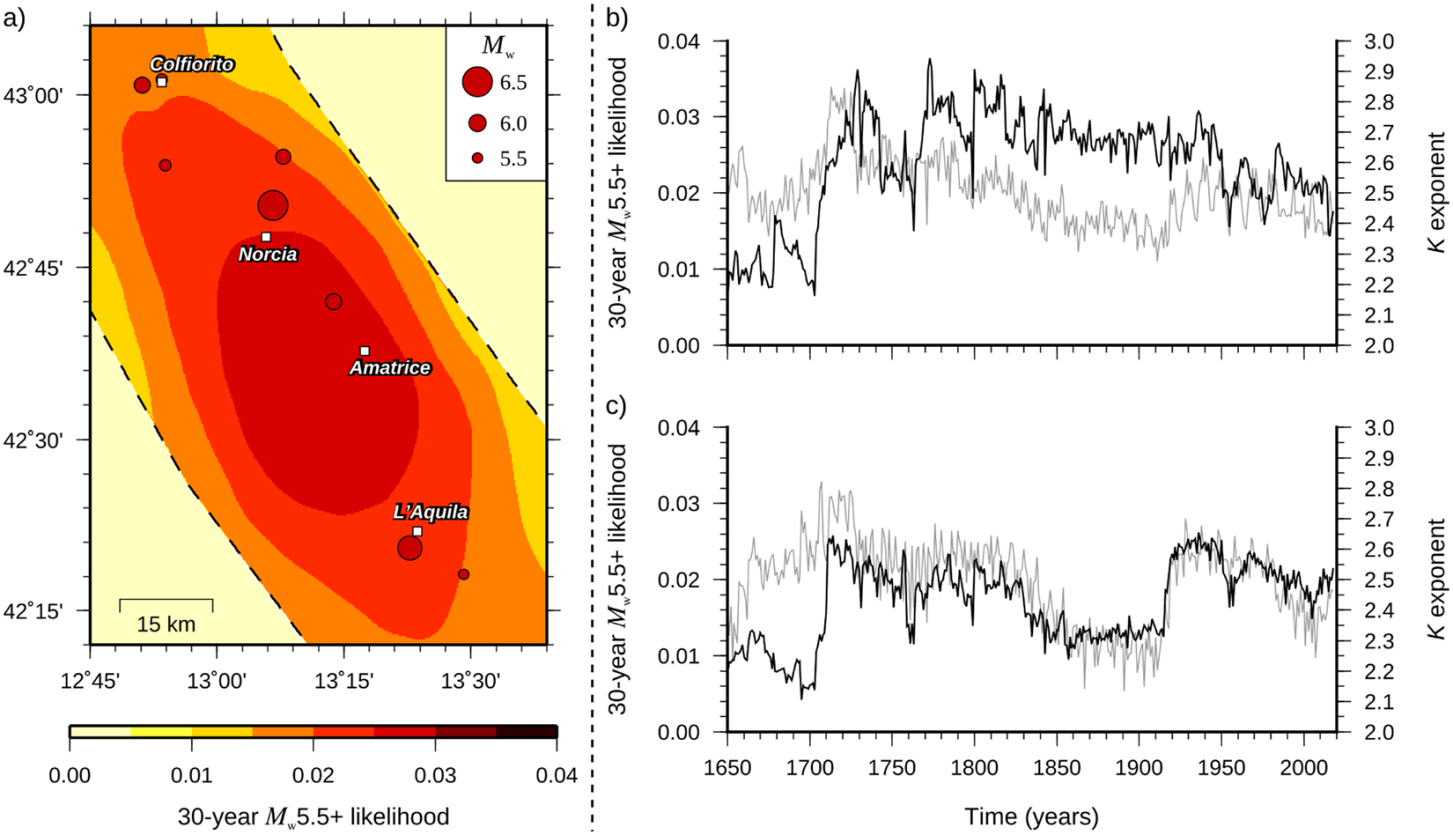
Map of the probability of occurrence of one or more events with magnitude above 5.5 per cell of 0.04° × 0.04° during the 1987–2016 period (the relevant seismicity is superimposed) (**a**) and variation in time of the 30-year probability of exceeding an *M*_w_ = 5.5 earthquake (black line) for the sites of Norcia (**b**) and L’Aquila (**c**). The temporal variation of the *K* exponent (gray line) is superimposed in Fig. 7b,c.

As an example, Fig. 7a presents a retrospective forecast map for the 1987–2016 period that is based on the data until December 1986. Precisely, it shows the geographic distribution of the 30-year probability of exceeding a magnitude 5.5 earthquake (within cells of 0.04° × 0.04°) in the Central Apennines since January 1987. Beyond the geographic distribution of probabilities, which are higher between Norcia and L’Aquila, where there have been two of the three largest seismic sequences of the last 30 years, the map is presented as a sort of simple retrospective test of our model for long-term forecasting. The period of 30 years is chosen as it represents the typical homeowner mortgage. Moreover, it is roughly the length of the instrumental catalog (the same as used in the examples shown in Fig. 4) that is used to examine the reliability of our predictions. Compatibly with the model, the benchmark catalog includes both foreshocks and aftershocks. To test the model, we compare the expected number of earthquakes estimated from the map with the observed ones. The average number of expected events is expressed by the equivalent 30-year Poisson rate; namely^44,49^:

8 $$\begin{array}{rcl}\lambda  & = & -\sum _{h=1}^{{N}_{c}}\mathrm{ln}(1-P{({M}_{{\rm{w}}} > 5.5|{\rm{\Delta }}t=30yrs)}_{h})\\  & = & -\sum _{h=1}^{{N}_{c}}\mathrm{ln}(1-P{({M}_{0}^{c}(n) > 2.24{\rm{E}}+17|n=30yrs)}_{h})\end{array}$$

where *N*_*c*_ indicates the number of cells of the grid used in the calculations.

Comparing the equivalent 30-year rate with the observed number of earthquakes shows good agreement (7.4 expected events vs. 8 actual). Note that the equivalent rate must not be confused with the actual Poisson rate obtained through catalog declustering. Removing dependent events significantly underestimates the observed rate (3 expected events vs. 8), thus affecting the seismic hazard.

Figure 7b,c finally shows the variation of the 30-year probability of exceeding a magnitude 5.5 earthquake for the sites of Norcia and L’Aquila. The probability of exceedance is obtained by increasing the ‘learning time’ since the beginning of the catalog (as with Fig. 1b,d, we kept the starting time fixed and moved the ending time forward 1 year at a time). Examining Fig. 7b,c shows that probabilities (black lines) jump following the 1703 and 1915 events, and subsequently tend to decrease at a slow rate that reflects closely the trend of *K* (gray lines), which, unlike the Hurst exponent, exhibits fluctuations with time.

## Discussion

It appears from the previous considerations that long-range dependence is an inherent feature of the seismic process. We have observed that the Hurst index is substantially space- and time-invariant, as it assumes a universal value of around 0.87, which is indicative of a memory process with marked long-range dependence. We have shown that variations in the *H* value are fictitious, and only occur if the seismic catalog is incomplete. It follows that one can observe such fictitious changes only if the observations are limited to a specific seismic episode (e.g., seismic sequence), or if they cover a short time interval within the entire historic record. In these cases, we have shown that *H* falls when the stress drops – that is, with the release of the seismic energy (foreshock, main shock, and aftershock stages) – and then climbs, to regain the basal level in the subsequent years (post-seismic stage).

Previous considerations motivated the development of a forecasting model that allows for persistency in the seismic process. As with many existing earthquake occurrence models, the model presented here has the advantage that it overcomes the assumption of seismicity as a Poisson process, which is commonly adopted in long-term forecasting. Hence, it allows for dependent events, such as foreshocks and aftershocks, thus avoiding the tricky phase of catalog declustering. In addition, its simple, yet effective, parameterization makes our model suitable for large-scale applications. In this study, the model is coupled with a smoothed gridded seismicity approach, which is used to determine the time series used in input (see Method section). This approach appears appropriate in areas that are characterized by frequent and diffuse seismicity, while the use of area sources (i.e., seismogenic zonations) might be preferable in regions where earthquakes are sparse and infrequent. Regionalization of seismicity into areas that are homogeneous with regard to seismic behavior and stress field is also advisable with smoothed seismicity, to thus account for the tectonic and geologic heterogeneities that can potentially influence the spatio-temporal distribution of earthquakes^50,51^ (i.e., the value of the *q* and *K* coefficients). Obviously, our model can also be adapted and used with fault sources.

As with all new methods, our forecasting model clearly deserves further testing, but the premise looks intriguing and the model promising, at the least for long-term (tens of years) probabilistic seismic hazard assessment. Encouraging results (see Supplementary Fig. S1) were also obtained for medium-term forecasting. Application of our model to forecast seismicity in the Italian Central Apennines in a 10-year time window (2010–2019) yielded results that are in perfect agreement with those obtained by Marzocchi *et al*.^52^ by averaging the results of four alternative skilled models. Furthermore, its applicability is not only limited to earthquake forecasting. Indeed, it can be extended to other disciplines that deal with self-similar processes, and particularly to other geohazards such as rainstorms, floods, landslides, avalanches, and tsunamis.

## Method

Gridded series of cumulated seismic moment were determined by application of the smoothed seismicity approach of Barani *et al*.^51^. Series were computed for each cell of a homogeneous grid of 0.04° spacing in latitude and longitude using the same parameterization adopted in Barani *et al*.^53^. Earthquake scalar moments were cumulated and smoothed iteratively by adding the contribution of 1 year (month or day, in the case of instrumental seismicity) at a time from the beginning of the catalog. An adaptive elliptical kernel was used when analyzing the instrumental seismicity, to avoid over-smoothing. In that case, the Kernel dimensions were estimated by applying the Wells and Coppersmith^54^ relations for rupture length (equal to the maximum semi-axis of the ellipse of smoothing) and width (equal to the minimum semi-axis). The Kernel dimensions were computed at each iteration as a function of the maximum earthquake magnitude observed until that time. No smoothing was applied on the Italian and worldwide series (Fig. 3).

Equation (1) and (2) were applied to each time series to compute the Hurst exponent. Specifically, each series was divided into an increasing number of (non-overlapping) intervals following a power-of-two scheme (i.e., *N* = 2^0^, 2^1^, 2^2^…, 2 ^*h*^) and the rescaled ranges (*R*/*S*)_*n*_ were computed. In more detail, the range *R*(*n*)_*k*_ in *k*-th sub-interval of length *n* is defined as:

9 $$R{(n)}_{k}=\,\max ({D}_{j,k})-\,\min ({D}_{j,k})$$

where ${D}_{j,k}={\sum }_{i=1}^{j}({X}_{i,k}-{E}_{i,k})$ for *j* = 1, …, *n* indicates the cumulative deviation of the *i*-th observation in the *k*-th window, *X*_*i,k*_, from the local trend (or profile), *E*_*i,k*_, here defined via polynomial regression. The average of the deviations from the local trend, *S*(*n*)_*k*_, is assessed here in terms of root-mean-square deviation, as:

10 $$S{(n)}_{k}=\sqrt{\frac{1}{n}\sum _{i=1}^{n}{({X}_{i,k}-{E}_{i,k})}^{2}}$$

For each interval, the degree of the detrending polynomial was automatically selected (in the range 1–5) by maximization of the coefficient of multiple determination, *R*^2^. According to Ośęwięcimka *et al*.^55^, polynomials of higher order were found to remove part of the fluctuation, leading to a slight reduction in *H*. On the other hand, constraining the polynomial degree to lower values was shown not to completely remove the trend^56^. However, we found that the choice of the profile has a minor impact on the results (less than 5%). Following different studies^17,57,58^, all analyses were made using a minimum number of 2^5^ intervals. For yearly historical time series, the maximum number of intervals was constrained to 2^6^ or 2^7^ to avoid windows with less than 10 observations. As the length of the series cannot be a multiple of *N*, the tail of the series can be left out of the analysis. In order not to disregard this part of the series, the procedure was repeated back and forth. Finally, to avoid overestimation of *H* due to small samples, the adjustment proposed by Peters (1994) was applied.

### Data availability

The authors declare that the materials and data used to produce the results presented in this manuscript will be made available promptly to the Editorial Board Members, Referees, and readers upon request.

## Electronic supplementary material

Supplementary Information
