# Supplementary material for: Long-range dependence in earthquake-moment release and implications for earthquake occurrence probability
Source: Sci Rep. 2018 Mar 28;8:5326. doi: 10.1038/s41598-018-23709-4 (PMC5871840; doi:10.1038/s41598-018-23709-4)
Supplement: Supplementary file 1 — Supplementary Information [file 41598_2018_23709_MOESM1_ESM.pdf]

Long-range dependence in earthquake-moment release and implications for earthquake occurrence probability

*Simone Barani[1] (e-mail: barani@dipteris.unige.it), Claudia Mascandola[2], Eva Riccomagno[3], Daniele Spallarossa[1], Dario Albarello[4], Gabriele Ferretti[1], Davide Scafidi[1], Paolo Augliera[2], and Marco Massa[2]

[1]Dipartimento di Scienze della Terra dell'Ambiente e della Vita, Università di Genova, Italy

[2]Istituto Nazionale di Geofisica e Vulcanologia, Sezione di Milano, Italy

[3]Dipartimento di Matematica, Università di Genova, Italy

[4]Dipartimento di Scienze Fisiche della Terra e dell'Ambiente, Università di Siena, Italy

Supplementary Note

The present note goes through the proof of equation (5) in the main article.

The application of Jensen's inequality to the logarithm of equation (1) in the main article (equivalently to taking the logarithm of the arithmetic mean-geometric mean inequality) leads to:

$$\log\left(\frac{1}{N}\sum_{k=1}^{N}\frac{R(n)_k}{S(n)_k}\right) \geq \frac{1}{N}\sum_{k=1}^{N}\log\left(\frac{R(n)_k}{S(n)_k}\right) \qquad \text{(S1)}$$

Equality in equation (S1) holds if and only if the $R(n)_k / S(n)_k$ values are equal.

Equation (S1) can also be written as:

$$\begin{aligned}\log\left(\frac{1}{N}\sum_{k=1}^{N}\frac{R(n)_k}{S(n)_k}\right) &\geq \frac{1}{N}\sum_{k=1}^{N}\left(\log R(n)_k - \log S(n)_k\right)\\ &= \frac{1}{N}\sum_{k=1}^{N}\log R(n)_k - \frac{1}{N}\sum_{k=1}^{N}\log S(n)_k \qquad \text{(S2)}\\ &= \log R(n) - \log S(n)\end{aligned}$$

where $\log R(n) = \frac{1}{N}\sum_{k=1}^{N}\log R(n)_k$ and $\log S(n) = \frac{1}{N}\sum_{k=1}^{N}\log S(n)_k$.

In logarithmic terms, equation (2) in the main article can be written as:

$$\log(R/S)_n = H\log n + p + \xi_{\log(R/S)_n} \qquad \text{(S3)}$$

where $\xi_{\log(R/S)_n}$ is the error term of the regression of $\log(R/S)_n$ on $\log(n)$ and $p = \log c$.

Substitution of equation (S3) in place of the left-hand side of equation (S2) leads to:

$$H\log n + p + \xi_{\log(R/S)_n} \geq \log R(n) - \log S(n) \qquad \text{(S4)}$$

From equations (5) and (6) in the main text, $\log R(n)$ can be predicted by $q + K\log n$. That is, $q + K\log n$ approximates the mean of the $\log R(n)_k$ values in equation (S2) and (S4). Substituting into equation (S4) yields:

$$H\log n + p + \xi_{\log(R/S)_n} \geq q + K\log n - \log S(n) \qquad \text{(S5)}$$

Rearranging equation (S5) leads to:

$$K \le H + \frac{\log S(n) + p - q + \xi_{\log(R/S)_n}}{\log n} \qquad \text{(S6)}$$

We quantified the discrepancy between the right-hand and left-hand sides in equation (S6) to obtain:

$$K \approx H + \left(\frac{\log S(n) + p - q}{\log n} \right) \qquad \text{(S7)}$$

In practice, $\log S(n)$ in equation (S7) can be predicted via linear regression; namely, assuming that also $S(n)$ follows a power law. Let J be the estimated slope and r the intercept.

Equation (S7) was verified for all nodes of the calculation grid used to produce the forecast map presented in Fig. 7 and for the Italian and worldwide time series presented in Fig. 3. For these time series and for three nodes of the calculation grid close to Norcia, Amatrice, and L'Aquila, Table S1 gives the values of the H, K, and J exponents and the p, q, and r intercepts. It emerges that unlike the Hurst exponent, K is region dependent. It is about 1.8 for the worldwide data set, 2.2 for the Italian one, and 2.4 for the study area.

Table S1 Power law coefficients H, K, J (slopes) and p, q, r (intercepts) estimated worldwide, for all of Italy, and for three sites in the Central Apennines.

Time series	Slope coefficient			Intercept coefficient		
	H	K	J	p	q	r
Worldwide	0.87 ± 0.03	1.81 ± 0.07	0.99 ± 0.07	-0.52 ± 0.07	18.95 ± 0.10	19.41 ± 0.09
Italian	0.84 ± 0.03	2.21 ± 0.14	1.39 ± 0.13	-0.51 ± 0.06	15.70 ± 0.19	16.19 ± 0.18
Norcia	0.88 ± 0.02	2.45 ± 0.23	1.60 ± 0.22	-0.55 ± 0.03	11.30 ± 0.31	11.79 ± 0.31
Amatrice	0.88 ± 0.02	2.42 ± 0.23	1.59 ± 0.23	-0.56 ± 0.04	11.32 ± 0.31	11.79 ± 0.31
L'Aquila	0.84 ± 0.02	2.44 ± 0.23	1.62 ± 0.22	-0.50 ± 0.03	11.15 ± 0.31	11.62 ± 0.31

Supplementary Fig. S1

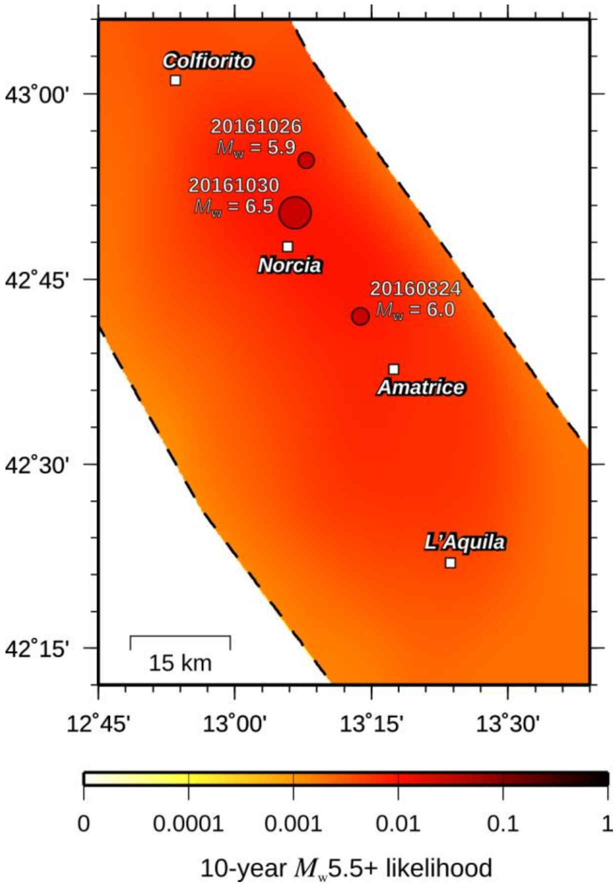

Figure S1 Map of the probability of occurrence of one or more events with magnitude above 5.5 per cell of 0.1° × 0.1° during the 2010-2019 period (earthquakes that occurred up to February 2017 are superimposed). For comparison purposes, the grid spacing and the color scale are the same as used by Marzocchi et al.[52] (see panel 3 of Fig. 10 therein).
